# Usability and Satisfaction Testing of Game-Based Learning Avatar-Navigated Mobile (GLAm), an App for Cervical Cancer Screening: Mixed Methods Study

**DOI:** 10.2196/45541

**Published:** 2023-08-08

**Authors:** Lindsey J Wanberg, Angela Kim, Rachel I Vogel, Karim Thomas Sadak, Deanna Teoh

**Affiliations:** 1 Medical School University of Minnesota Minneapolis, MN United States; 2 Laboratory of Cell and Developmental Signaling National Cancer Institute National Institutes of Health Frederick, MD United States; 3 Division of Gynecologic Oncology Masonic Cancer Center University of Minnesota Minneapolis, MN United States; 4 Division of Hematology Oncology Department of Pediatrics University of Minnesota Minneapolis, MN United States

**Keywords:** cancer screening, cervical cancer screening, cervical cancer, Game-based Learning Avatar-navigated mobile, health care app, mixed methods study, mobile health, mobile technology, Pap test, usability testing, young adult health care

## Abstract

**Background:**

Barriers to cervical cancer screening in young adults include a lack of knowledge and negative perceptions of testing. Evidence shows that mobile technology reduces these barriers; thus, we developed a web app, Game-based Learning Avatar-navigated mobile (GLAm), to educate and motivate cervical cancer screening using the Fogg Behavioral Model as a theoretic guide. Users create avatars to navigate the app, answer short quizzes with education about cervical cancer and screening, watch videos of the screening process, and earn digital trophies.

**Objective:**

We tested ease of use, usefulness, and satisfaction with the GLAm app among young adults.

**Methods:**

This mixed methods study comprised a qualitative think-aloud play interview session and a quantitative survey study. Participants were cervical cancer screening–eligible US residents aged 21 to 29 years recruited through social media. Qualitative study participants explored the app in a think-aloud play session conducted through videoconference. Data were analyzed using directed content analysis to identify themes of ease of use, usefulness, and content satisfaction. Qualitative study participants and additional participants then used the app independently for 1 week and completed a web-based survey (the quantitative study). Ease of use, usefulness, and satisfaction were assessed using the validated Technology Acceptance Model and Computer System Usability Questionnaire adapted to use of an app. Mean (SD) scores (range 1-7) are presented.

**Results:**

A total of 23 individuals participated in one or both study components. The mean age was 25.6 years. A majority were cisgender women (21/23, 91%) and White (18/23, 78%), and 83% (19/23) had at least some secondary education. Nine participants completed the think-aloud play session. Direct content analysis showed desire for content that is concise, eases anxiety around screenings, and uses game features (avatars and rewards). Twenty-three individuals completed the quantitative survey study. Mean scores showed the app was perceived to be easy to use (mean score 6.17, SD 0.27) and moderately useful to increase cervical cancer screening knowledge and uptake (mean score 4.94, SD 0.27). Participants were highly satisfied with the app (mean score 6.21, SD 1.20).

**Conclusions:**

Survey results showed participants were satisfied with the app format and found it easy to use. The app was perceived to be moderately useful to inform and motivate cervical cancer screening; notably, the screening reminder function was not tested in this study. Qualitative study results demonstrated the app’s ability to ease anxiety about screening through demonstration of the screening process, and brevity of app components was favored. Interpretation of results is limited by the predominantly cisgender, White, and educated study population; additional testing in populations which historically have lower cervical cancer screening uptake is needed. A modified version of the app is undergoing efficacy testing in a randomized clinical trial.

## Introduction

Adherence to cervical cancer screening guidelines among eligible individuals aged 21-29 years decreased between 2005 and 2016 [1]. Barriers to screening in young adults include lack of knowledge or awareness, negative perceptions of testing, and systemic barriers [2]. Mobile health (mHealth) technology has the potential to improve knowledge outside of the clinical encounter [3-5]. A meta-analysis of various mHealth platforms showed technology was associated with increased uptake of cancer screening, with the largest effect for cervical cancer screening (pooled OR 1.71, 95% CI 1.34-2.19) [6]. A scoping review concluded mHealth interventions may be an effective way to reach individuals in need of screening and improve cervical cancer screening uptake [7]. These results suggest mHealth has a role in cervical cancer screening uptake, but interventions are varied and most often comprise SMS text messages, telephone calls, or social media messaging, and less often mobile apps. The purpose of this study was to test the ease of use, usefulness, and satisfaction of the Game-Based Learning Avatar-Navigated Mobile (GLAm) app, designed to increase knowledge and awareness of cervical cancer and screening [2] and elucidate the screening process.

## Methods

### GLAm App Development

The GLAm app was developed in collaboration with the University of Minnesota Education Technology Innovations team (Multimedia Appendix 1). The objective of the app is to educate and motivate guideline-adherent cervical cancer screening among average-risk individuals aged 21-29 years. The Fogg Health Behavior Model was used as a theoretic guide [8], and the app design was informed by results from testing an app previously developed by our group [5]. The current app was tailored to a younger population and incorporated more gamified aspects. The content was informed by a literature review of barriers to cervical cancer screening in young adults and a focus group study conducted by the study team. A user-created avatar navigates the app, which comprises a single education module in testing mode. The app uses a multiple-choice quiz format to test knowledge and to provide information on each topic. Some explanations included short videos (eg, how a Pap test is performed). Trophies are awarded for app registration, avatar creation, education module completion, videos watched, and logging completed cervical cancer screening (Multimedia Appendix 1). The app is programmed to send SMS text message reminders for the next recommended screening test, but this feature could not be tested in this study. The app includes links to additional cervical cancer screening information.

### Recruitment

Participants were recruited through a social media (Facebook and Instagram)–promoted message over a 2-week time period. Inclusion criteria were being female, being at average risk for cervical cancer, being aged 21-29 years, and residing in the United States, per self-report. Interested participants submitted a form with their contact information on the internet through the secure Research Electronic Data Capture (REDCap) web app [9] managed by the University of Minnesota Clinical and Translational Science Institute. Potential study participants were then contacted by the study team by email or telephone, per their preference. The anticipated number of usability problems was small given the simple design of the GLAm app, and sample size was determined through standard practices for data obtained through think-aloud methodologies [10,11]. Goal accrual for the qualitative study was 6 participants (maximum 9). The goal accrual for the quantitative survey study was 12 participants (maximum 24) and included the qualitative study participants.

### Ethics Approval

The University of Minnesota Institutional Review Board approved the study (#STUDY00010234). Participants provided written informed consent and Health Insurance Portability and Accountability Act (HIPAA) authorization form electronically through REDCap [12], and participants were emailed a PDF of the signed consent. All study data were deidentified. Each study participant was assigned a unique study number, and the link between the study number and participant identifiers was maintained in a REDCap database that was separate from any study data. Survey data were stored in REDCap [9]. Other deidentified study data, including videos of the think-aloud play, were stored in a HIPAA-compliant cloud-based Box Secure Storage file managed by the University of Minnesota. Study participants in the think-aloud play were instructed in advance to remove their name from the Zoom screen and were provided with their study number to use as their username for the app so that their identity was not visible at any point during the think-aloud play. All data collection and storage plans were approved by the University of Minnesota Health Information Privacy and Compliance Office as part of the institutional review board review and approval process. Participants were compensated with a US $50 gift card for participation in the think-aloud play interview and a US $25 gift card for 1-week app review and survey completion.

### Mixed Methods

To test the usability, usefulness, and satisfaction of the GLAm app, we followed a mixed methods approach, first conducting a qualitative think-aloud play interview session to examine usability and to identify and revise any problematic components of the app. This was followed by a separate quantitative survey study to describe acceptability and satisfaction with the app. Both studies are described in detail below, and integration of the components is illustrated in Multimedia Appendix 2.

### Qualitative Study

Initial usability testing was performed using think-aloud play through videoconferencing (Zoom). After receiving standardized verbal instructions, the participant shared their screen and voiced their thoughts while interacting with the GLAm app. The think-aloud play was observed by the study’s principal investigator (DT) and research assistant (LJW). Study staff did not interact with the participants during think-aloud play except for a prerecorded prompt (“keep talking”) if the participant was silent for 15 seconds. App components identified as problematic by the first group of think-aloud play participants (n=5) were changed before testing of the last 4 participants and the quantitative study: (1) correction of a misspelled word in 1 quiz answer explanation; (2) rewording of a quiz question for better clarity; and (3) reorganization of the opening screen to make avatar creation more visible. Sessions were video recorded and auto-transcribed using videoconferencing technology. Transcripts were reviewed and corrected, and nonverbal observations were added. Data were analyzed using directed content analysis to identify major themes of ease of use, usefulness, and content satisfaction. A member of the study team (LJW) read and coded transcripts, with a second senior team member (KTS) participating in the coding. The team reviewed quotes, codes, interpretations, and themes that ultimately evolved into thematic labels, concepts, and relationships. Once thematic saturation was achieved, thematic patterns were evaluated for similarities and differences that additionally refined coding and identified the final themes.

### Quantitative Study

Participants interacted with the app independently for 1 week and then completed a survey on acceptability and satisfaction. Acceptability was assessed using the validated Technology Acceptance Model [13], with questions adapted to the use of an app. Satisfaction was measured using the validated Computer System Usability Questionnaire [14], adapted to the use of an app. Surveys were completed on the internet using REDCap [9]. Mean (SD) scores (minimum score 1 and maximum score 7) are reported.

## Results

### Qualitative Study

#### Overview

The demographics of the 9 participants are detailed in Tables 1 and 2. All identified as non-Hispanic, Latina, or Latino and (8/9) 89% identified as White. All regions of the country were represented. All were cisgender people, and 8 had at least some secondary education. The mean think-aloud play time was 14.9 (SD 5.2) minutes. We identified 3 major themes.

**Table 1:** Participant demographics in qualitative study (think-aloud play; n=9).

Participant	Gender	Age (years)	Race or ethnicity^a^	Education
1	Cisgender female	24-26	White	Secondary school
2	Cisgender female	24-26	Native Hawaiian or Pacific Islander	Graduate school
3	Cisgender female	27-29	White	Graduate school
4	Cisgender female	21-23	White	High school or General Educational Development test
5	Cisgender female	27-29	White	Secondary school
6	Cisgender female	27-29	White	Graduate school
7	Cisgender female	21-23	White	Secondary school
8	Cisgender female	21-23	White	Secondary school
9	Cisgender female	24-26	White	Graduate school

^a^Ethnicity was assessed separately from race.

**Table 2:** Participant demographics in quantitative study (survey). The study population for the quantitative study (n=23) also included survey responses from the 9 participants in the qualitative study.

Characteristics		Values
Age (years), mean (SD)		25.6 (2)
**Gender, n (%)**		
	Cisgender female	21 (91)
	Nonbinary	1 (4)
	Missing	1 (4)
**Race or ethnicity^a^, n (%)**		
	Asian	2 (9)
	Black	2 (9)
	Hawaiian or Pacific Islander	1 (1)
	White	18 (79)
	Hispanic or Latina	2 (9)
**Education, n (%)**		
	High school or General Educational Development test	4 (17)
	Secondary	9 (39)
	Graduate	10 (44)

^a^Ethnicity was assessed separately from race.

#### Theme 1: Ease of Use—Educational Content Must Be Concise and Easily Digestible

Several participants preferred time-efficient and easy-to-comprehend educational content. Many participants enjoyed learning through the question-and-answer format of the game, and conciseness was favored (Table 3; participant 1). One participant expressed frustration with the quiz format (Table 3; participant 3), and frequently skipped reading informative explanations. Participants were satisfied with visual learning methods (Table 3; participants 1, 2, and 7). The Pap test video was 2.5 minutes long, and 2 participants did not watch it completely, expressing, “I know the gist of how that goes” and “I think I’ve seen enough of that video, I kind of get the concept.” (participants 1 and 6).

**Table 3:** Summary of major themes with sample quotes.

Theme	Sample Quotes
1. Educational content must be concise and easily digestible.	I’m just gonna watch the video instead of reading. (Participant 1) I’m a very big visual person so I think the pictures and the videos are really helpful. (Participant 2) This is kind of going on a long time. I feel like you could have shown me like a one page with this information, and then I already have it as opposed to the questions. (Participant 3)
2. Content that eases anxiety around screenings is most helpful.	I do really like that there is a video on this because I feel like my first time having gone to the OB/GYN, I had no idea what they were doing. And I was in tears, so it’s really nice that, you know, for younger people, there is a video of kind of what’s going on. I know it’s not the same as being there, but this is nice. (Participant 5) [The app is] kind of a fun way to get more information and feel a little bit better about making that appointment. Still nervous to do it, but I know that I need to do it. This is a sign that I need to do it. (Participant 4)
3. Gamification was the main driver of user satisfaction.	I got another trophy! I really like that, I think it’s cute. (Participant 6) Let’s see what I can get trophies for… I am curious what other trophies I can get. Let’s see... I am going to make an avatar too, that sounds fun. Oh cute! Let’s see... I like that they are little animals and not, like, my face. That’s kind of cute. (Participant 7)

#### Theme 2: Usefulness—Content That Eases Anxiety Around Screening Is the Most Helpful

A total of 5 participants mentioned negative feelings about screening. The screening was described as “scary,” “painful,” and “uncomfortable” (participants 4-6). One participant admitted she had been avoiding the Pap test “like the plague” (participant 4). Participants also expressed confusion and anxiety about interpreting screening recommendations and results: “[Receiving results can be] super confusing, super scary, especially if you have never received an abnormal Pap test” (participant 5).

Several participants found the Pap test video to be a helpful resource for reducing anxiety (Table 3; participants 4 and 5). One participant suggested adding more content to ease anxiety around screening: “I think something that would be nice to be included is that there are different size speculums that you can even request at an OB/GYN appointment...comfort is so important...including information about that would be really nice” (participant 5).

#### Theme 3: Satisfaction—Gamification Was the Main Driver of User Satisfaction

Participants enjoyed the gamified aspects of the app, such as the avatar, trophies, and app response to correct or incorrect quiz answers. Participants often cheered if they got a lesson question correct. All participants who created an avatar mentioned something positive about this feature (Table 3; participant 5). Participants found the animal avatars to be cute and better at providing anonymity than human avatars or profile pictures. Seven participants responded positively to receiving trophies (Table 3; participants 6 and 7). Only 1 participant expressed a negative opinion: “I’m going to be honest that I personally don’t care about trophies...it really doesn’t make a difference to me at all” (participant 9).

### Quantitative Study

A total of 24 people (including 9 think-aloud participants) enrolled in the quantitative study, but 1 person did not submit responses to any survey questions and was omitted from data analysis (Tables 1 and 2). Summary demographics include the 9 individuals who participated in both the qualitative and quantitative studies. Most participants identified as White and cisgender people; 1 participant identified as nonbinary gender; and all census race categories were represented except American Indian and Alaskan Native. Participants were highly educated (Tables 1 and 2).

The mean perceived ease of use score was 6.17 (SD 0.27), indicating the app was easy to use (Table 4). The mean perceived usefulness score was 4.94 (SD 0.22), suggesting the app would be moderately useful in increasing adherence to cervical cancer screening guidelines. Overall satisfaction with the app was high, with a mean satisfaction score of 6.12 (SD 0.40).

**Table 4:** Survey results (quantitative study; n=23).

			Scores, mean (SD)
**Perceived ease of use^a^**			6.17 (0.27)
	Learning to use the GLAm app was easy for me.	6.35 (1.27)	
	I find it easy to get the GLAm app to do what I want it to do.	5.91 (1.47)	
	My interaction with the GLAm app was clear and understandable.	6.21 (1.20)	
	I find the GLAm app to be flexible to interact with.	5.78 (1.28)	
	It would be easy for me to become skillful at using the GLAm app.	6.34 (0.98)	
	I find the GLAm app easy to use.	6.43 (0.84)	
**Perceived usefulness^a^**			4.94 (0.27)
	Using the GLAm app would enable me to get factual information about cervical cancer screening more quickly.	5.00 (1.57)	
	Using the GLAm app would improve my ability to follow cervical cancer screening guidelines.	4.70 (1.58)	
	Using the GLAm app would increase my ability to get on-time cervical cancer screening.	5.04 (1.52)	
	Using the GLAm app would enhance my ability to get cervical cancer screening.	4.83 (1.47)	
	Using the GLAm app would make it easier for me to know when to get cervical cancer screening.	5.30 (1.36)	
	I would find the GLAm app useful in guiding my personal cervical cancer screening practice.	4.78 (1.50)	
**Satisfaction^b^**			6.12 (0.40)
	Overall, I am satisfied with how easy it is to use the GLAm app.	6.70 (0.64)	
	It was simple to use the GLAm app.	6.56 (0.73)	
	I could effectively complete the tasks using the GLAm app.	6.17 (1.23)	
	I was able to complete the tasks quickly using the GLAm app.	6.21 (1.24)	
	I was able to efficiently complete the tasks using the GLAm app.	6.21 (1.20)	
	I felt comfortable using the GLAm app.	6.56 (0.66)	
	It was easy to learn to use the GLAm app.	6.48 (0.73)	
	I believe I could become knowledgeable about cervical cancer screening quickly using the GLAm app.	5.52 (1.75)	
	Whenever I made a mistake in the GLAm app, I could recover easily and quickly.	5.35 (1.61)	
	The information (eg, on-screen messages) provided with the GLAm app was clear.	6.13 (1.32)	
	It was easy to find the information I needed.	5.70 (1.79)	
	The information provided for the GLAm app was easy to understand.	6.26 (1.32)	
	The information was effective in helping me complete the tasks and scenarios/The information was effective in helping me complete the tasks.	5.90 (1.55)	
	The organization of information on the GLAm app screens was clear.	6.00 (1.35)	

^a^Assessed using the Technology Acceptance Model [13].

^b^Assessed using the Computer System Usability Questionnaire [14].

## Discussion

### Principal Results

Study participants were satisfied with the GLAm app, the education provided, and the gamified components of the app. The app was perceived as easy to use. The perceived usefulness of the app to increase cervical cancer screening uptake was more moderate, but the qualitative study results showed that it was useful for decreasing anxiety and fear associated with the Pap test. Notably, the SMS text message reminder for screening was not tested in this study. The brevity of app components was reinforced by participants who skipped reading educational explanations or truncated the 2.5-minute videos.

### Comparisons With Previous Work

Similar to the results from our study, multiple studies of electronic game–based learning have shown high appeal and satisfaction with the game format [15-17]. While many reviews of mHealth studies to increase preventative health behavior have shown positive results, these studies combine multiple interventions, predominantly SMS text messaging, telephone calls, and social media messaging, with a smaller number of studies evaluating mHealth apps [7,18]. The gamified Fight HPV app comprises puzzles demonstrating complex scientific concepts such as how the immune system and various health behaviors impact the risk of human papilloma virus (HPV) infection and cancer [19]. A retrospective cohort study showed that downloading the app was associated with a greater than 2-fold increase in cervical cancer screening compared to age- and cancer screening history-matched controls [19]. This demonstrates the potential effectiveness of an app to increase cervical cancer screening but does not directly address barriers such as fear of the screening process itself, which was addressed in this app through text explanations, pictures, and videos.

### Strengths and Limitations

Usability and satisfaction were evaluated using both qualitative and quantitative methodologies using validated survey questions. However, while scores on the Technology Acceptance Model predict usage, there is no score threshold to indicate “usefulness,” limiting the interpretation of results. Limitations also include selection bias inherent to the social media recruitment strategy [20,21]: parameters (age, geographic region, budget, and keywords) for promoted messages are entered into the social media platform, and a proprietary algorithm is used to target users who see the platform. Users self-select how they respond to the advertisement, which in our study resulted in a predominantly White and highly educated population; the app needs to be further tested in populations who historically have the lowest screening coverage. Last, the efficacy of the app to increase cervical cancer screening uptake was not tested in this study, and distribution of an effective app remains a challenge. During the qualitative portion of the study, 1 participant suggested the distribution of the app to adolescents through school programs.

### Future Directions

To address the challenge of distribution of a health app, we decided to test the effectiveness of the app to increase preventative health behaviors within a childhood cancer survivorship clinic. The survivorship clinic was selected as this provided a way to distribute the app in a health care setting in which participants were not already presenting for the target preventative care. A revised version of the app using the same format and visual features tested for usability and satisfaction was created to encourage HPV vaccination among survivors of childhood cancer. A change in focus from cervical cancer screening to HPV vaccination was chosen to target the greatest number of participants in the survivorship clinic (upper age limit of 27 years; all genders). This is also an important aspect of survivorship care which is often not emphasized [22,23]. The efficacy of the app in increasing HPV vaccination initiation and completion is currently being tested in a randomized clinical trial.

## Appendix

Multimedia Appendix 1 Screenshots of the Game-Based Learning Avatar-Navigated Mobile (GLAm) app, including the (A) GLAm app dashboard; (B) avatar creation feature; (C) lesson content; (D) screening log.

Multimedia Appendix 2 Graphic representation of integration of the qualitative and quantitative components of the study.

## Abbreviations

GLAm: Game-based Learning Avatar-navigated mobile
HIPAA: Health Insurance Portability and Accountability Act
HPV: human papilloma virus
mHealth: mobile health

## Data Availability

The data sets generated during and/or analyzed during this study are available from the corresponding author on reasonable request.
