# Supplementary figures and images for: Usability and Satisfaction Testing of Game-Based Learning Avatar-Navigated Mobile (GLAm), an App for Cervical Cancer Screening: Mixed Methods Study
Source: JMIR Form Res. 2023 Aug 8;7:e45541. doi: 10.2196/45541 (PMC10445170; doi:10.2196/45541)

## Slide 1
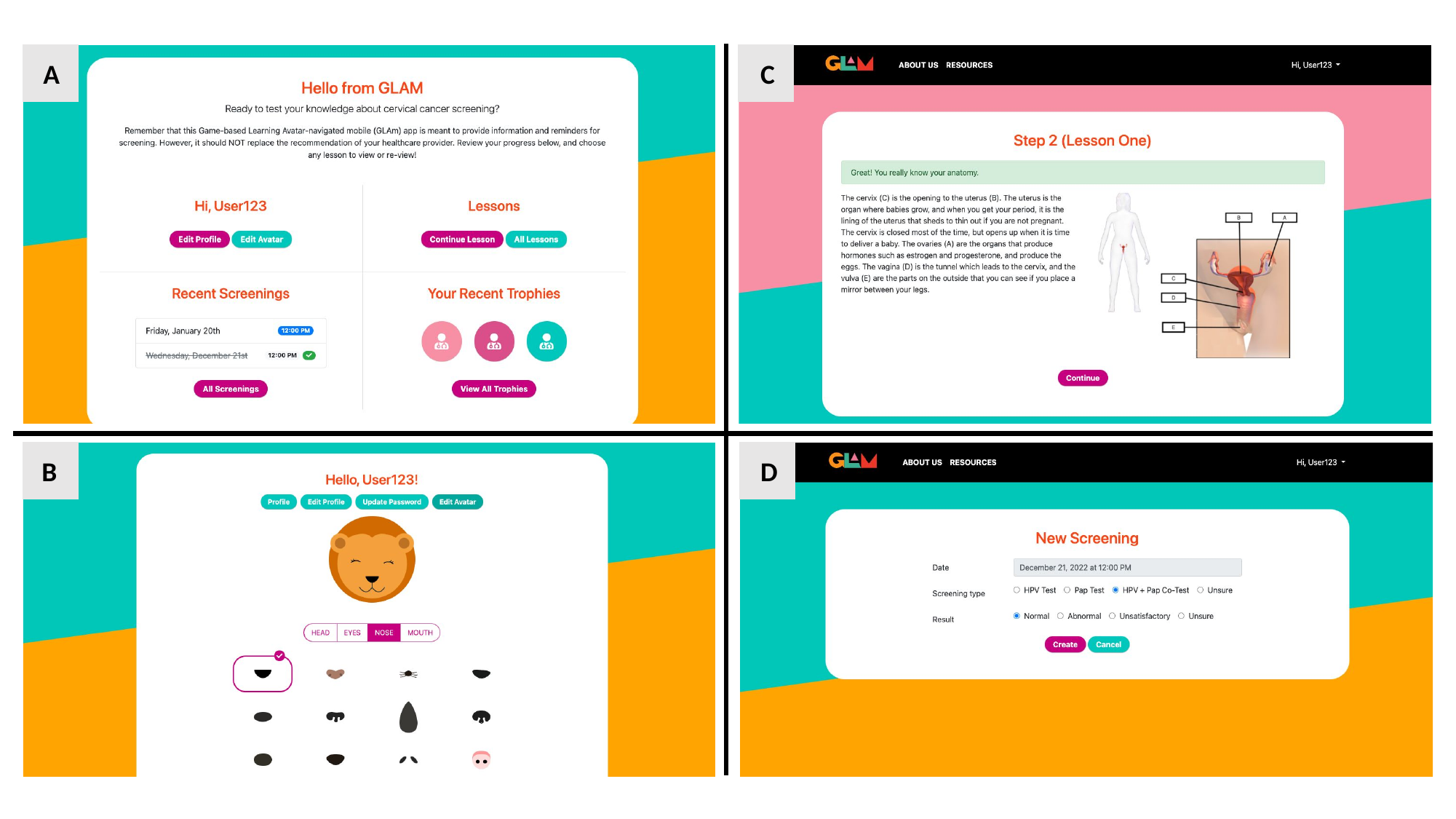

A
C
B
D

Supplement: Multimedia Appendix 1 [file formative_v7i1e45541_app1.pptx]

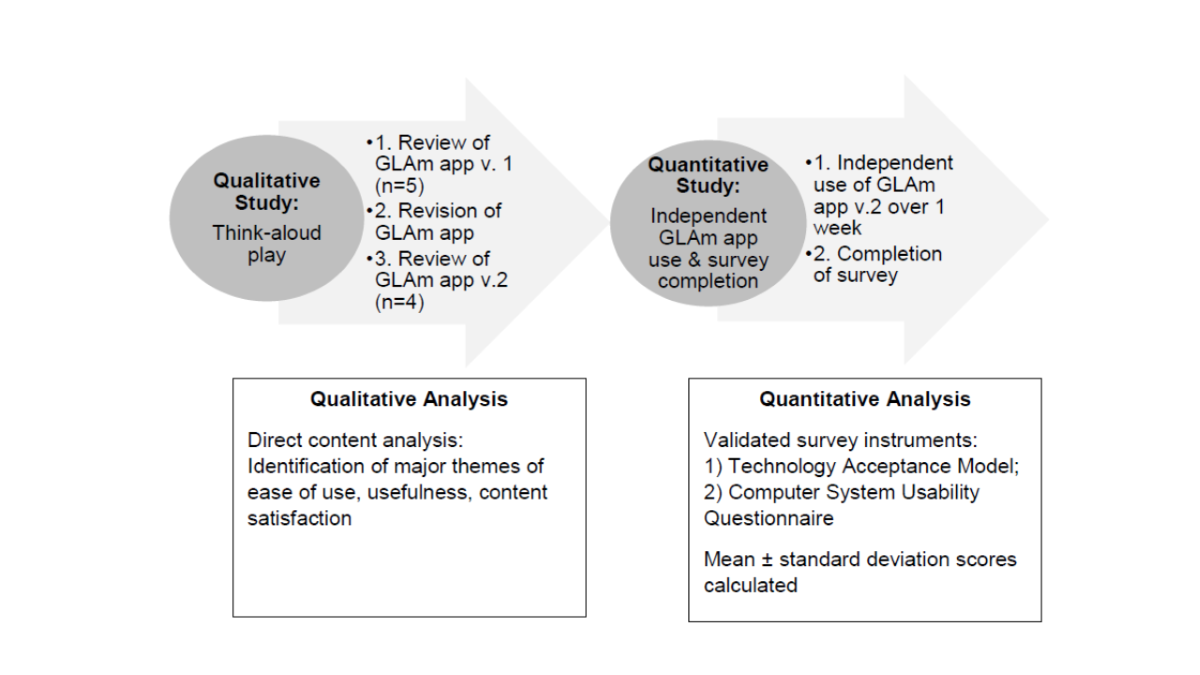

Supplement: Multimedia Appendix 2 [file formative_v7i1e45541_app2.png]
